# Moving Toward Telehealth Surveillance Services for Toddlers at Risk for Autism During the COVID-19 Pandemic

**DOI:** 10.3389/fpsyt.2020.565999

**Published:** 2020-11-26

**Authors:** Eugenia Conti, Natasha Chericoni, Valeria Costanzo, Roberta Lasala, Alice Mancini, Margherita Prosperi, Raffaella Tancredi, Filippo Muratori, Sara Calderoni, Fabio Apicella

**Affiliations:** ^1^Department of Developmental Neuroscience - Istituto di Ricovero e Cura a Carattere Scientifico Fondazione Stella Maris, Pisa, Italy; ^2^Department of Clinical and Experimental Medicine, University of Pisa, Pisa, Italy

**Keywords:** autism spectrum disorders (ASD), coronavirus disease 2019 (COVID-19), public mental health, neurodevelopment, early identification, telehealth

## Abstract

Since 2016, the project “Early Bird Diagnostic Protocol for Autism Spectrum Disorders (ASD)” funded by the Italian Ministry of Health has been operative at IRCCS Fondazione Stella Maris (FSM), Pisa (IT), with the main aim of developing early age-specific diagnostic protocols by longitudinally enrolling two different populations at risk for ASD: (i) toddlers with older siblings with ASD (FR) and (ii) toddlers referred by a child psychiatrist or pediatrician for suspected ASD (CR). On January 30, 2020, when the World Health Organization declared the outbreak of coronavirus disease 2019 (COVID-19), 136 patients (85 FR; 51 CR; 93 males; 43 females) had been enrolled in the project with 324 completed time points and 64 still missing. Considering both the huge psychological burden on families with toddlers at risk for ASD during the lockdown and the longitudinal studies reporting the positive “surveillance effect” in terms of a better outcome in at-risk toddlers, our priority has been to maintain regular contact and support to enrolled families. To do this, the research team, being authorized for smart-working research activities, has set up a detailed remote surveillance protocol (RSP). The RSP includes three online interviews and one online video registration of parent–child play. In the current community case study, the authors report the telehealth procedure and discuss possible future directions in developing remote assessment and new evaluation modalities for ecological parent–child play video recordings in at-risk populations. Hopefully, the surveillance protocol will further improve our ability to detect risk and activate early tailored intervention.

## Introduction

With an estimated prevalence of one in 87 children aged 7–9 years in Italy (1), autism spectrum disorders (ASD) are increasingly perceived as a public health priority, with a significant individual, familial, and societal burden, both emotional and economic. The phenotypic expression and the detrimental impact of ASD could be mitigated through early identification and intervention prior to the emergence of full-blown symptoms. Current literature, in fact, suggests that (i) it is possible to detect ASD starting from 14 months of age in at least a certain proportion of children (2); (ii) very young children benefit from early intervention (EI), especially when parents are actively involved in the rehabilitation process (3); and (iii) intervention should be initiated as soon as possible, when signs of ASD risk appear (4). Based on these three assumptions, since 2016 the project “Early Bird Diagnostic Protocol for Autism Spectrum Disorders” (EARLY BIRD; NET-2013-02355263-3) funded by the Italian Ministry of Health has been operative at IRCCS Fondazione Stella Maris (FSM), Pisa (IT), a tertiary-care University hospital that receives patients from all over Italy. This project aimed to develop, through longitudinal clinical monitoring and planned time points, age-specific diagnostic protocols able to detect (i) risk for ASD at 12 months of age; (ii) provisional diagnosis of ASD at 18 months of age; and (iii) stable diagnosis according to the diagnostic criteria of the DSM-5 (5) at 24 months of age. In addition, a clinical follow-up is scheduled at 30 months of age, to confirm the diagnosis or exclude it definitively. Children whose evaluations suggest a diagnosis of autism are referred to their local Child and Adolescent Mental Health Services (CAMHS) for early intervention.

A team of psychologists and child psychiatrists with many years of clinical and research experience in the ASD Unit of the FSM are in charge of the EARLY BIRD surveillance protocol of two different at-risk populations: (i) toddlers with older siblings with ASD (familial risk toddlers: FR), who are vulnerable to ASD and, more broadly, to psychiatric and neurodevelopmental disorders (6, 7) and (ii) toddlers clinically referred (CR) for suspected ASD by a child psychiatrist or pediatrician.

From September 2016 to January 2020, 136 children (85 FR; 51 TR; 93 males; 43 females) were enrolled in the study (see Table 1). A total of 106 subjects (FR: 58; CR: 48) have completed the diagnostic protocol, while 30 (FR: 27; CR: 3) are still being monitored. As part of the EARLY BIRD project, we completed 324 time points of the children enrolled, while 64 are still missing (5 at 12 months; 11 at 18 months; 18 at 24 months; 30 at 30 months).

**Table 1:** Number of subjects at each time point (TP) are divided into completed (COM) and planned (PLA) protocol.

	**Subjects**		**Total TP**		**12 m**		**18 m**		**24 m**		**30 m**	
	**COM**	**PLA**	**COM**	**PLA**	**COM**	**PLA**	**COM**	**PLA**	**COM**	**PLA**	**COM**	**PLA**
CR	48	3	106	3	4	0	29	0	41	0	32	3
FR	58	27	218	61	57	5	61	11	55	18	45	27
TOT	106	30	324	64	61	5	90	11	96	18	77	30

*CR, Clinical referral; FR, familial risk; TOT, Total*.

In its original form, the EARLY BIRD surveillance protocol was conceived as a 2-day assessment of cognitive functioning, adaptive functioning, and social and communicative functioning, through standardized tests and interviews [Griffiths Mental Developmental Scales-ER (8), Vineland 2 (9), ADOS-2 (10), ADI-R (11)]. In addition, other information on each child's development was collected through parental questionnaires and clinical interviews (12–23) (see Table 2).

**Table 2:** The questionnaires administered to parents at each time point of the EARLY BIRD surveillance protocol.

	**Time points (age of the child)**			
	**12 months**	**18 months**	**24 months**	**30 months**
M—CHAT (12)		✓		
RBS—R (13, 14)		✓	✓	✓
CBCL 1.5-5 (15)		✓	✓	✓
ITSEA (16)	✓	✓	✓	✓
FYI (17)	✓			
CDIs (18)	✓	✓	✓	✓
LUI (19)		✓	✓	✓
EMQ (20)	✓	✓	✓	✓
PSI III (21)	✓	✓	✓	✓
QUIT (22)	✓	✓	✓	✓
SP (23)	✓	✓	✓	✓

*CBCL 1.5-5, Child Behavior Checklist; CDIs, MacArthur–Bates; Communicative Development Inventories; EMQ, Early Motor Questionnaire; FYI, First Year Inventory; ITSEA, Infant–Toddler Social and Emotional Assessment; LUI, Language Use Inventory; M-CHAT, Modified Checklist for Autism in Toddlers; PSI III, Parenting Stress Index, Third edition; QUIT, Italian Questionnaires of Temperament; RBS-R, Repetitive Behavior Scale-Revised; SP, Sensory Profile*.

On January 30, 2020, the World Health Organization declared the outbreak of coronavirus disease 2019 (COVID-19) a public health emergency of international concern (24).

Emerging evidence reports the hard situation in which parents have been put during this unpredictable stressful situation, potentially impairing their ability to be supportive caregivers (25). Indeed, parents, especially those with young children, have to suddenly cope with closure of the kindergartens, worries over health and finances, the necessity for quick adaptation to “smart-working” at home, isolation, lack of support from grandparents, and bans on going to parks or other public spaces. Parents of toddlers at risk for ASD are even more exposed to stress, experimenting with concern that their child might have atypical development can impact family well-being and increase parents' risk of depression, ruminative thought, anxiety, or other types of psychological distress (26). In addition, stressful factors can interfere with early dyadic interaction between at-risk infants and their caregivers and, ultimately, play a detrimental role in children's longer-term social functioning and outcome (27). Some interesting suggestions about coping with young children with ASD in “stay at home period” are reported in Narzisi (28).

Coping difficulties may have even greater repercussions in families with infants who have an older sibling already diagnosed with ASD. Indeed, it is reported that caregivers of children with ASD have a higher risk of decreased family cohesion, depression, anxiety, somatic complaints, and burnout in comparison to caregivers of children with other developmental disabilities (29–32).

For all these reasons, our priority has been to maintain regular contact and support the families whose child was being followed in his developmental trajectory by the multidisciplinary team, in order to continue the active surveillance of toddlers at risk for ASD during the COVID-19 crisis. Indeed, a recent review of longitudinal studies on FR toddlers reported that the prospective follow-up strategy improves their developmental outcome, creating a sort of “surveillance effect” through which parents, who have the possibility to talk regularly about their child's development with clinical experts, can learn new strategies to interact with their high-risk infants, reducing, in turn, their symptom severity (33).

Despite technological tools and facilities (such as smartphones, tablets, PC, and wi-fi connections) spread and used among populations, there is little use for telehealth-based assessments of clinical conditions, including ASD, as recently systematically reviewed in Dahiya et al. (34). Obviously, the final goal consists of reaching the same percentage of success of the “in-person” assessment.

In a recent contribution, Juarez et al. (35) analyzed the accuracy of a remote diagnostic assessment for high-risk infants in the second–third year of life (the diagnosis was later confirmed with an *in-person* visit), using a remote video-analysis of a screening tool—the STAT (36–38). Despite that this diagnostic procedure succeeded in 62% of cases and the procedure was well-accepted by parents, this was still dependent on the “physical” presence of a trained person who could administer the screening tool. On the contrary, Smith et al. (39) validated a novel remote procedure for ASD symptom assessment, where parents were instructed to elicit and record specific target behaviors in different scenarios, comparing it with an *in-person* assessment. Results indicated a high agreement (88.2%) between the two modalities as well as high sensitivity (84.9%) and high specificity (94.4%). Following the same line, Sutantio et al. (40) found a good level of agreement (82%) between parents' video recording, based on a given specific protocol, in three different home settings and a direct assessment conducted by an expert clinician.

In another study (41), Fusaro and colleagues interestingly examined the potential of applying the ADOS-G (42) protocol to unstructured homemade videos collected via the YouTube platform. They found that the ADOS-G-based video analysis achieved a classification accuracy of 96.8, 94.1% of sensitivity, and 100% of specificity.

All these studies revealed that parents are capable of collecting appropriate examples of target behaviors of their children, especially when properly guided, and that these homebased procedures might improve the ecological validity of a diagnostic assessment (43). For this reason, we decided to adapt our original surveillance protocol to a remote procedure, aiming firstly to support families during the pandemic period and to provide, when necessary, feedbacks and advice on how to support child development or helping families to create a fast link to the local CAMHS.

Our efforts have been focused on instructing families on how to collect as much meaningful information as possible and to record an appropriate video recording of children's behavior. For this reason, differently from the previous studies on this topic, we have decided to be virtually present during the video recording, to promptly encourage parents to acquire appropriate examples of target behaviors.

This “perspective” paper describes how the protocol, already in use within the EARLY BIRD project, has been rethought and readjusted to be used remotely following the guidelines of health authorities, local governments and the World Health Organization in the COVID-19 era.

### Remote Surveillance Protocol

The research team consists of three psychologists (V.C., F.A., and N.C.) and two child neurologists and psychiatrists (A.M. and R.T.). Due to the lockdown, all researchers have been authorized to continue activities in smart-working.

The team meets via video conference twice a week to discuss ongoing updates and issues, sharing the documents and materials for the remote surveillance protocol RSP within a shared folder on the cloud.

The RSP includes three online interviews and one online video registration of parent–child interaction in a playful setting, for a total of four online sessions. A preliminary telephone call is planned to inform families about the adapted telematic procedure and accept or decline participation. Afterward, caregivers who decide to participate in the remote evaluation fill in written informed consent procedures in compliance with institutional review board standards. Once informed consent has been acquired by the research team, parents are administered the following questionnaires by research assistants.

Detailed instructions on how to prepare the play area for the video recording are also provided. A minimum level of structure (e.g., in the selection of toys) is required in order to obtain informative video material, sufficiently comparable across children (44, 45).

#### First Online Session

The psychologist and the child psychiatrist meet the parents online to record 15 min of parent–child play. Parents are encouraged to resolve any doubts about the setup of the video registration before starting, taking advantage of the researchers' help. A picture of the setting is taken in order to facilitate the replicability of the video recording at future time points. Researchers follow the recording in “mute option,” intervening only if necessary, to ensure the visibility of both parent and child.

In the days following the first session, the video is viewed and discussed by the researchers, focusing on those behaviors, included in the ADOS-2 Toddler Module (46) diagnostic algorithm, that does not need to be elicited by an adult: overall quality of the social overtures, eye contact, use of gestures to communicate, language level and prosody, play variety, joint attention behaviors, facial expressions, presence of unusual sensory interests, presence of repetitive movements and presence of restricted and repetitive patterns of behaviors and interests, and presence of other problematic behaviors. At the end of the discussion, a clinical hypothesis on the risk for ASD is made, as a starting point to be further examined during the following two sessions.

#### Second Online Session

An anamnestic interview or an anamnestic update is collected. Researchers also discuss with parents any concerns and information that emerged from questionnaires. Parents are asked to report recent auxological parameters (height, weight, and head circumference) as well as other general health status information. Vineland-II interview (9) with one parent is also carried out.

#### Third Online Session

The Socio-emotional Bayley III (47) interview is administered to parents along with a non-structured interview focused on age-appropriate behavioral risk signs for ASD or with the ADI-R (48) (in the case of toddlers older than 24 months). Further information on the developmental level of the infant is collected through a non-structured interview based on the developmental milestones of play skills.

After the third session, the clinical-research team meets to discuss the information collected from the interviews and parental questionnaires, in order to decide if the child needs additional assessments to further investigate any causes for concern, which may have arisen. If not, the child will be evaluated again at his next time-point.

#### Fourth Online Session

The last session with parents includes feedback on the evaluations and discussion about developmental issues (if present). The appointment for the next time-point is then scheduled. Parents are also asked to compile a questionnaire to collect feedback on the remote procedure they have used including suggestions for improving the service.

If any kind of diagnosis or a consistent risk for ASD is hypothesized at one of the planned time points, the child's parents will be informed. At the same time, the local CAMHS will be contacted in order to start as soon as possible an early treatment tailored to specific strengths and challenges of the child.

**Figure 1:**
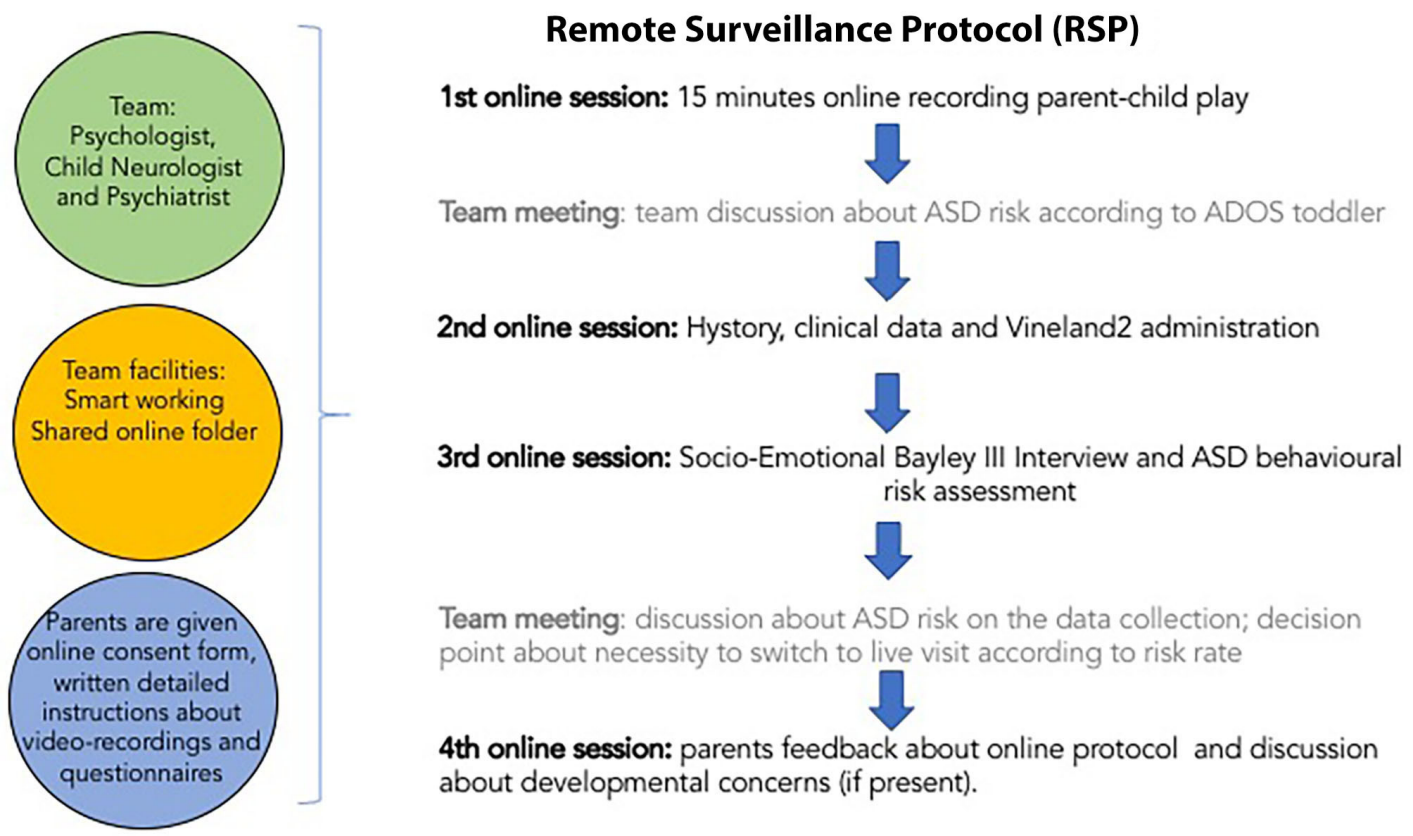
Schematic representation of the remote surveillance protocol (RSP).

A schematic representation of RSP is reported in Figure 1.

## Considerations

Lockdown, which is often an unpleasant experience due to isolation, uncertainty about health and economical status with extensive effects on the general population (49), could have even worse consequences in those families with concerns about their child's development, thus forcing health workers to find out alternative remote strategies to keep in touch with them.

Preexisting evidence was already available about remote modality application in the ASD field either in assessment or in rehabilitation programs (34, 50), and during the COVID-19 emergency, the role of remote assistance in children with special needs has been even more stressed (51, 52).

The adaptation of the Autism Surveillance protocol reported here was systematized by the research team within the first few weeks of the Italian lockdown in order to do the following:

(1) maintain regular contact and support families involved in the longitudinal surveillance of their FR or CR toddler's development;
(2) evaluate the feasibility of an online surveillance protocol for toddlers at risk for autism and obtain evaluations at the time-points established by the project; and
(3) assess the level of compliance and satisfaction on the part of the families with regard to this new telematic protocol.

The current procedure emerged from multiple discussions among professionals with long experience in the field of early typical and atypical development. The main aim was to be able to perform an assessment, which was as structured and standardized as possible. One of the first challenges met was the impossibility to conduct a remote assessment of ASD symptoms and cognitive development using standardized tests such as the ADOS-Toddler and the Griffiths Mental Developmental Scales. To assess the child's spontaneous social behavior, a video depicting the child in interaction with the parent was collected. One of the most debated points regarding the procedure for the video registration was the level of structure. If, on the one hand, we wanted to collect clinically useful information, we did not want to stress parents by asking them to try to elicit particular behaviors (e.g., response to joint attention and imitation skills) as if they were clinicians. For this reason, we chose to ask parents to play with their child as they would naturally, but requested them to provide a specific selection of toys among those available in their house in order to elicit different levels of play and standardize the setting across different children. Despite these limitations, we are convinced that this could represent a way to experiment new procedures, potentially helpful in the future, to reach those patients who have difficulties getting to the hospital as has already been achieved in other countries (53).

Furthermore, the video recordings will also be helpful to assess parent–child interaction in their natural environment (54, 55). In order to obtain information on the children's development, we chose to investigate their level of play skills. For this purpose, we created a dedicated checklist that could be administered to parents during video-conference interviews.

Finally, we encouraged parents to comment on their experience of the telematic adaptation of the procedure and to share their level of satisfaction and perceived efficacy of the different web-platform appointments, highlighting any critical issues and providing suggestions where possible. It will provide researchers with precious feedback that will facilitate future improvements in remote procedures.

The preliminary feedback we have received since the start of the remote surveillance protocol suggests, in the majority of cases, a positive response to our proposal by the families, who have appreciated the possibility to keep in touch with the research team and to share any developmental concerns about their child. In addition, the telehealth procedure we have implemented allows families to avoid traveling to the FSM (they arrive from all over Italy), saving in this way time, money, time off from work, and potentially stressful experience for both parents and child (e.g., due to travel meltdowns and sensory overload that could occur in the child).

## Future Directions

Active surveillance of infants at risk for ASD should continue during COVID-19 social distancing restrictions in order to start an intensive intervention during early sensitive periods (56) for toddlers identified with atypical development. To this aim, we implemented the current protocol intended to provide telehealth assessment in at-risk populations.

We are fully aware that our telehealth assessment is not exhaustive and that the lack of remote standardized tests for ASD diagnosis could limit the ability to detect early risk signs of the disorder, especially with milder cases, leading us to be cautious in our diagnostic conclusions.

Unquestionable advantages of this procedure include (i) better access, especially for families with transportation and childcare challenges; (ii) the possibility of removing job-related absence as well as cost of the travel; and (iii) the opportunity to observe the child in their naturalistic environment and accordingly to provide more family-centered recommendations.

Conversely, extensive use of telehealth certainly needs the following: (i) appropriate knowledge about data security and data protection rules; (II) the use of user-friendly platforms that can be accessible by all caregivers regardless of their technological abilities or economic status; and (iii) the reduction of regulatory barriers that interfere with reimbursement for services provided via telehealth.

To overcome these difficulties, we used free platforms that are easily downloadable from any online store and do not require payment; we have also created a video tutorial to make downloads for parents. Through the online video registration, we have also avoided the telematic transmission of data by parents, reducing difficulties and risks deriving from this. Moreover, the informed consent contains a detailed section on data protection according to European regulation (GDPR 2016/679 (Prot. 4/2018 PO) and approved by our institutional Data Protection Officer.

In conclusion, in spite of negative health, social, and economic consequences, the current COVID-19 crisis could represent an opportunity to reorganize child mental health care by introducing innovative approaches through telehealth, thus paving the way for broader access and more efficient use of available public resources (57). Future investigations should accurately compare remote surveillance to face-to-face evaluations (e.g., via randomized, controlled trials) in order to shed light on the efficacy, large-scale feasibility, and cost-effectiveness of online procedures, with the final aim of developing evidence-based guidelines for a virtuous coexistence of virtual and in-person assessment (58).

## Data Availability Statement

The raw data supporting the conclusions of this article will be made available by the authors, without undue reservation.

## Ethics Statement

The studies involving human participants were reviewed and approved by Pediatric Ethical Committee of Tuscany region at Meyer Children's Hospital. Written informed consent to participate in this study was provided by the participants' legal guardian/next of kin.

## Author Contributions

NC, VC, AM, RT, and FA conceived and created the protocol. FM supervised all the phases of the study. EC and SC wrote the paper. NC, VC, RL, AM, MP, FM, RT, and FA critically reviewed the manuscript. All authors contributed to the article and approved the submitted version.

## Conflict of Interest

The authors declare that the research was conducted in the absence of any commercial or financial relationships that could be construed as a potential conflict of interest.
